# Anisotropic magnetotransport and extremely large magnetoresistance in NbAs_2_ single crystals

**DOI:** 10.1038/s41598-018-24823-z

**Published:** 2018-04-23

**Authors:** G. Peramaiyan, Raman Sankar, I. Panneer Muthuselvam, Wei-Li Lee

**Affiliations:** 1 Institute of Physics, Academia Sinica, Taipei, 10617 Taiwan; 2 Center for Condensed Matter Sciences, National Taiwan University, Taipei, 10617 Taiwan; 3 Department of Materials Science, Central University of Tamil Nadu, Neelakudi, Thiruvarur, 610005 Tamil Nadu India

## Abstract

We report the extremely large magnetoresistance and anisotropic magnetoresistance in a non-magnetic semimetallic NbAs_2_ single crystal. Unsaturated transverse XMR with quadratic field dependence has been observed to be ~3 × 10^5^ % at 2 K and 15 T. Up to 12.5 K, clear Shubnikov de Haas (SdH) quantum oscillations were observed from which two distinct Fermi pockets were identified. The corresponding quantum electronic parameters such as effective cyclotron mass and Dingle temperature were obtained using Lifshitz-Kosevich formula. From the field dependent Hall resistivity at 2 K, carrier concentrations *n*_*e*_(*n*_*h*_) = 6.7691 (6.4352) × 10^25^ m^−3^ and mobilities *μ*_*e*_ (*μ*_*h*_) = 5.6676 (7.6947) m^2^ V^−1^ s^−1^ for electrons (e) and holes (h) were extracted using semiclassical two-band model fitting. We observed large anisotropic magnetoresistance about 84%, 75%, and 12% at 0.75 T and 6 K for three different orientations *γ*, *θ* and *ϕ*, respectively, similar to that in several topological semimetallic systems. Magnetic properties of NbAs_2_ are similar to the case of graphite, without any phase transition in the temperature range from 5 K to 300 K.

## Introduction

Exploration of novel states of quantum matter with exotic physical phenomena is one of the new frontiers in condensed matter physics. Unusual transport properties such as large magnetoresistance (MR) not only provide signatures of unique states of matter but also play a vital role in device applications such as magnetic field sensors, random access memories, hard drives, spintronic devices, etc^1–3^. The unsaturated large magnetoresistance with quadratic field (H^2^) dependence, transverse and longitudinal linear magnetoresistance in nonmagnetic semimetals are unusual phenomena, and its origin is under debate in condensed matter physics. In some semimetals such as NbSb_2_^4^, LaSb^5^ and LaBi^6^, the origin of large unsaturated MR with H^2^ is attributed to the electron-hole compensation. On the other hand, the electron-hole compensation with H^2^ of MR and linear MR at intense high fields are observed in the topological semimetals such as a Dirac semimetal, ZrSiS^7^ and a Weyl semimetal TaAs^8^, but its origin is different from those aforementioned materials^9^. Among the family of nonmagnetic semimetallic systems, NbAs_2_ crystallizes in monoclinic with inversion center (C12/*m*1)^10^, and is demonstrated to exhibit large MR with H^2^ dependence^10–13^. First principle calculations revealed that the NbAs_2_ system possesses four types of Fermi surfaces^11^. It is reported that the NbAs_2_ single crystal shows unsaturated large transverse MR about 8000 at 9 T and 1.8 K^11^, 8800 at 9 T and 2 K^12^, 1000 at 14 T and 2.5 K^13^ and ultra-high mobility of the order of 10^4^−10^5^ cm^2^ V^−1^ s^−1^, and its origin is attributed to electron-hole compensation. The field induced XMR with metal-insulator-like cross-over behavior followed by a resistivity plateau has been observed in a nonmagnetic semimetallic system NbAs_2_^12,13^, where nontrivial Berry phase^13^ and negative longitudinal MR^11^ have also been observed. However, the detailed angle dependent magnetoresistance study will help to understand the anisotropic properties of NbAs_2_, which has not been fully studied. In this work, we report a systematic study of anisotropic magnetoresistance (AMR) in NbAs_2_ crystal. Large AMR in NbAs_2_ may be linked to the non-trivial Berry phase of topological systems. High magnetic field transport measurement in the *I*⊥*H* geometry shows the large unsaturated parabolic MR. The results of fitting with a semiclassical two-band model reveal electron-hole compensation with temperature dependent mobility in NbAs_2_.

## Results and Discussion

**Figure 1:**
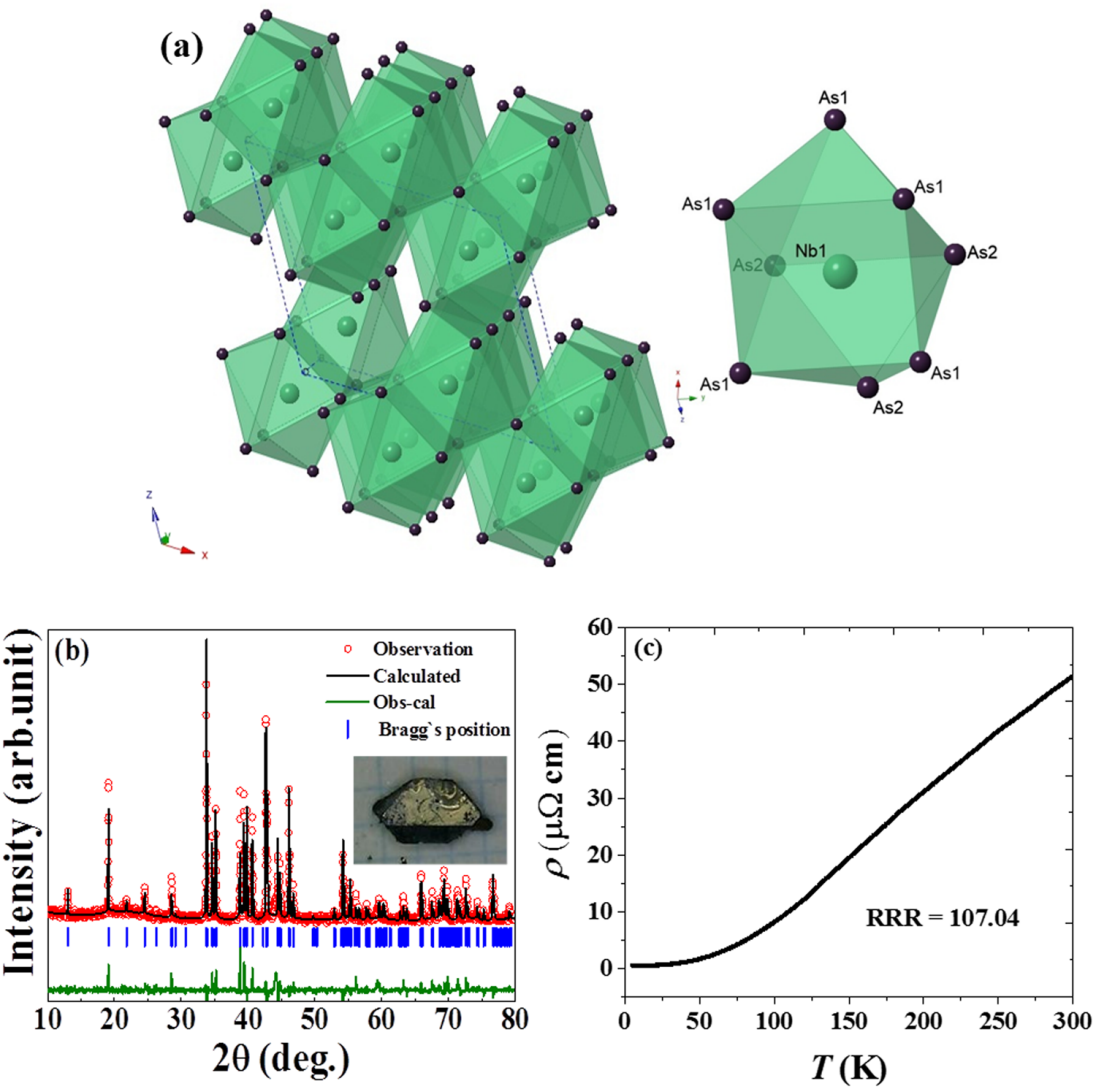
(**a**) Crystal structure of NbAs_2_. The inset shows the triangular prism of NbAs_2_ where a Nb (Nb1) atom is bounded by six As (As1, As2) atoms and two As atoms lie outside the rectangular faces. (**b**) Powder X-ray diffraction (XRD) pattern and Rietveld refinement results of for pulverized NbAs_2_ single crystals. The inset shows the as-grown single crystal of NbAs_2_. (**c**) shows the temperature dependence of resistivity with metallic profile, RRR about 107.04.

**Figure 2:**
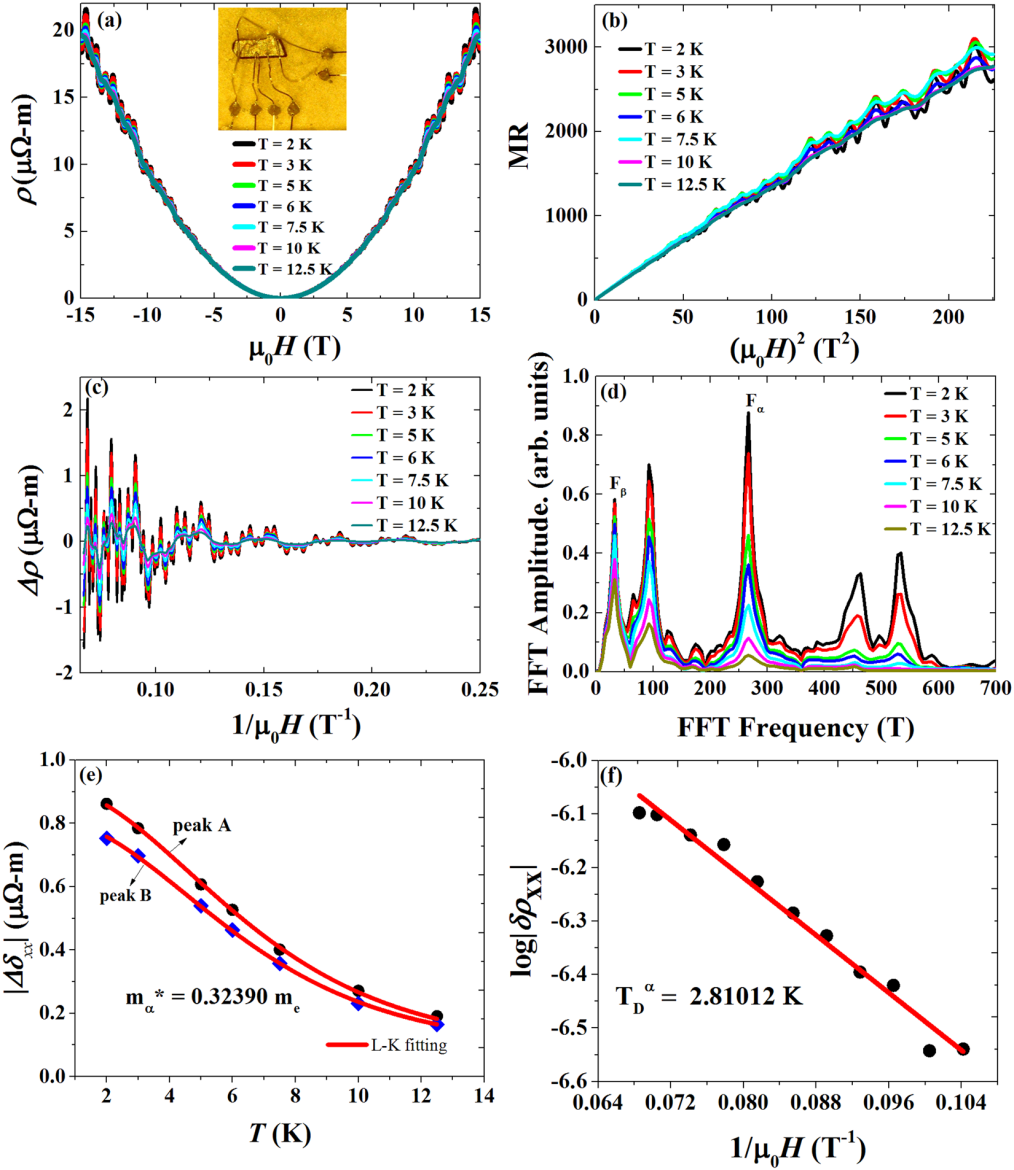
(**a**) Field dependence of resistivity *ρ* (*H*, *T*) of sample-1 along *I*⊥*H* at various temperatures showing clear SdH quantum oscillations. The insets show the measurement geometry (left) and six-probe geometry for the simultaneous measurement of **ρ**_xx_ and **ρ**_xy_ (right). (**b**) It shows the quadratic field dependence of magnetoresistance MR = [*ρ*(*H*) − *ρ*(0)]/*ρ*(0)] of sample −1. (**c**) Total resistivity oscillatory patterns *Δρ*_*xx*_ as a function of inverse magnetic field (1/*Mμ*_*0*_*H*) at various temperatures for the *I*⊥*H* geometry. (**d**) FFT spectrum of quantum oscillations showing two distinct peaks at F_α_ = 266 T and F_β_ = 32 T as well as their harmonics for various temperatures. (**e**) Temperature dependence of oscillation amplitude at fixed magnetic field for the observed Fermi pocket. Peak A&B represent the positions of resistivity oscillatory amplitudes. The solid line is the L-K fitting, used to extract the effective cyclotron mass and Dingle temperature. (**f**) It shows the log |*∆ρ*_*xx*_| vs *T* plot at 3 K, and linear fitting yields the Dingle temperature.

NbAs_2_ crystallizes in a monoclinic system with the centrosymmetric space group of C12/*m*1. It belongs to a larger family of transition metal dipnictides *MPn*_2_ (*M* = V, Nb, Ta, Cr, Mo, and W, *Pn* = P, As and Sb), which is found to crystallize in OsGe_2_ structure type. In the NbAs_2_ crystal structure (as shown in Fig. 1(a) and its inset), each Nb (Nb1) atom is bounded by six As (As1, As2) atoms and two As atoms lie outside the rectangular faces. Figure 1(b) shows the Rietveld refinement of the X-ray powder diffraction results (Bruker D8) using Cu*-K*_*α*_ radiation for the pulverized crystalline sample of NbAs_2_. The inset of Fig. 2(a) shows the as-grown single crystals of NbAs_2_. The refined lattice parameters, *a* = 9.3560 (2) *Å*, *b* = 3.3828 (1) *Å*, *c* = 7.7966(2) *Å*, and *β* = 119.440(15)°, are in good agreement with those reported in the literature^10^.

Figure 2(a) shows the plot of resistivity as a function of magnetic field at various temperatures measured in the *I*⊥*H* configuration. The data obtained by sweeping the magnetic field from 15 T to −15T were then symmetrized using *ρ*(*H*) = [*ρ*(*H*) + *ρ*(−*H*)]/2. NbAs_2_ exhibits quite large magnetoresistance (MR) at low temperatures with a strong Shubnikov de Haas (SdH) quantum oscillation and quadratic field dependence as shown in Fig. 2(b). The MR percentage calculated from [(*ρ*(*H*) − *ρ*(0))*/ρ*(0)] × 100%, reaches 303,200% at 2 K without any signature of saturation in a field of 15 T. It is observed that the MR of NbAs_2_ is very sensitive with respect to sample quality, since sample-1 shows MR about 115,200% at 2 K and 9 T with a residual resistivity ratio (RRR = ρ_300K_/ρ_3K_) of 107.04 (Fig. 1(c)), whereas the MR of sample 2 shows 170,800% with a RRR of about 110.49 as shown in Fig. S1. The RRR of NbAs_2_ crystals attest to the good metallicity and quality of the grown crystals, which is comparable to that reported for the Weyl semimetal NbP (RRR = 115)^9^, higher than those of TaAs (RRR = 49)^8^ and NbAs (RRR = 72)^14^, but lower than less than that previously reported for NbAs_2_ (RRR = 222, and 317)^11,12^ crystals. The unsaturated MR behavior of NbAs_2_ is similar to the semimetals WTe_2_^15^ and NbSb_2_^4^. The large and unsaturated MR of NbAs_2_ is higher than that for the semimetals NbSb_2_ (MR = 1.3 × 10^5%^ at 2 K and 9 T)^4^, LaBi (MR = 0.38 × 10^5^% at 2 K and 14 T)^6^, the Dirac semimetals ZrSiS^7^ (MR = 1.4 × 10^5^% at 2 K and 9 T) and Cd_3_As_2_ (MR = 1.6 × 10^5^% at 2.5 K and 15 T)^16^. It is comparable with that recently reported for NbAs_2_ (8 × 10^5%^ at 9 T at 1.8 K^11^, 1 × 10^5%^ at 14 T at 2.5 K^13^), the topological semimetal LaSb (9 × 10^5^% at 9 T and 2 K)^17^ and the Weyl semimetal candidates NbP (8.5 × 10^5%^ at 9 T at 1.85 K)^9^ and NbAs (MR = 2.3 × 10^5^% at 9 T and 2 K)^14^. In order to analyse the SdH quantum oscillations, the second order polynomial smoothed background was subtracted from the field dependent resistivity, *ρ*(*H*). Figure 2(c) shows the total oscillatory pattern (*Δρ*) with obvious quantum oscillations starting from 4 T as a function of inverse magnetic field (1/*μ*_0_*H*) for (*I*⊥*H*) geometry. From the fast Fourier transformation spectrum (FFT) as shown in Fig. 2(d), two major peaks are observed at *F*_*α*_ = 266 T and *F*_*β*_ = 32 T as well as their harmonics, where α and β are denoted as high and low frequency peaks, respectively. Since the amplitude of total oscillation (*Δρ*_*xx*_) seems to show complex periodic behavior, FFT frequency filtering was used to extract the respective oscillation patterns denoted as (*δρ*) for the observed frequency of 266 T to estimate the cyclotron effective mass (m*) and Dingle temperature (*T*_*D*_) as shown in Fig. S2(b). From the Onsager relation $F=({\varphi }_{0}/2{\pi }^{2}){A}_{F}$, where the *A*_*F*_ is the extremal Fermi surface cross-sectional area perpendicular to the field, *F* is the frequency of the oscillation, and ${\varphi }_{0}$ is the magnetic flux quantum. The Fermi surface cross sections are calculated to be 25.3 × 10^−3^ Å^−2^ and 3.04 × 10^−3^ Å^−2^ for 266 T and 32 T, respectively. The total oscillatory pattern (*∆ρ*) can be expressed based on the Lifshitz-Kosevich (L-K) formalism^18^

1 $${\rm{\Delta }}\rho (T,B)=\exp [-X({T}_{D},B)]\frac{X(T,B)}{\sinh \,[X(T,B)]}\Delta \rho ^{\prime} $$

where ${\rm{\Delta }}\rho ^{\prime}$ is the oscillatory component without damping, and $X(T,B)=2{\pi }^{2}{k}_{B}{Tm}^{\ast }/\hslash eB$. Here, m* refers to the effective cyclotron mass, and *T*_*D*_ is the Dingle temperature. The temperature dependence of *δρ* is fitted well with the L-K formula as shown in Fig. 2(e). The fitting results yield the effective cyclotron mass ${m}_{\alpha }^{\ast }=0.323\pm 0.00090{m}_{e}$, where *m*_*e*_ is the electron rest mass. Figure 2(f) shows the fitting results of the respective *δρ* for various inverse fields (1*/μ*_0_*H*) at a fixed temperature of 3 K, which yields the Dingle temperature ${T}_{D}^{\alpha }=2.810\pm 0.004\,K$. From the Dingle temperature, the single particle scattering rate is calculated to be ${\tau }_{s}=\frac{\hslash }{2\pi {k}_{B}{T}_{D}}$ = 4.35 × 10^−13^ s. The obtained results of NbAs_2_ are consistent with previous studies^11–13^.

**Figure 3:**
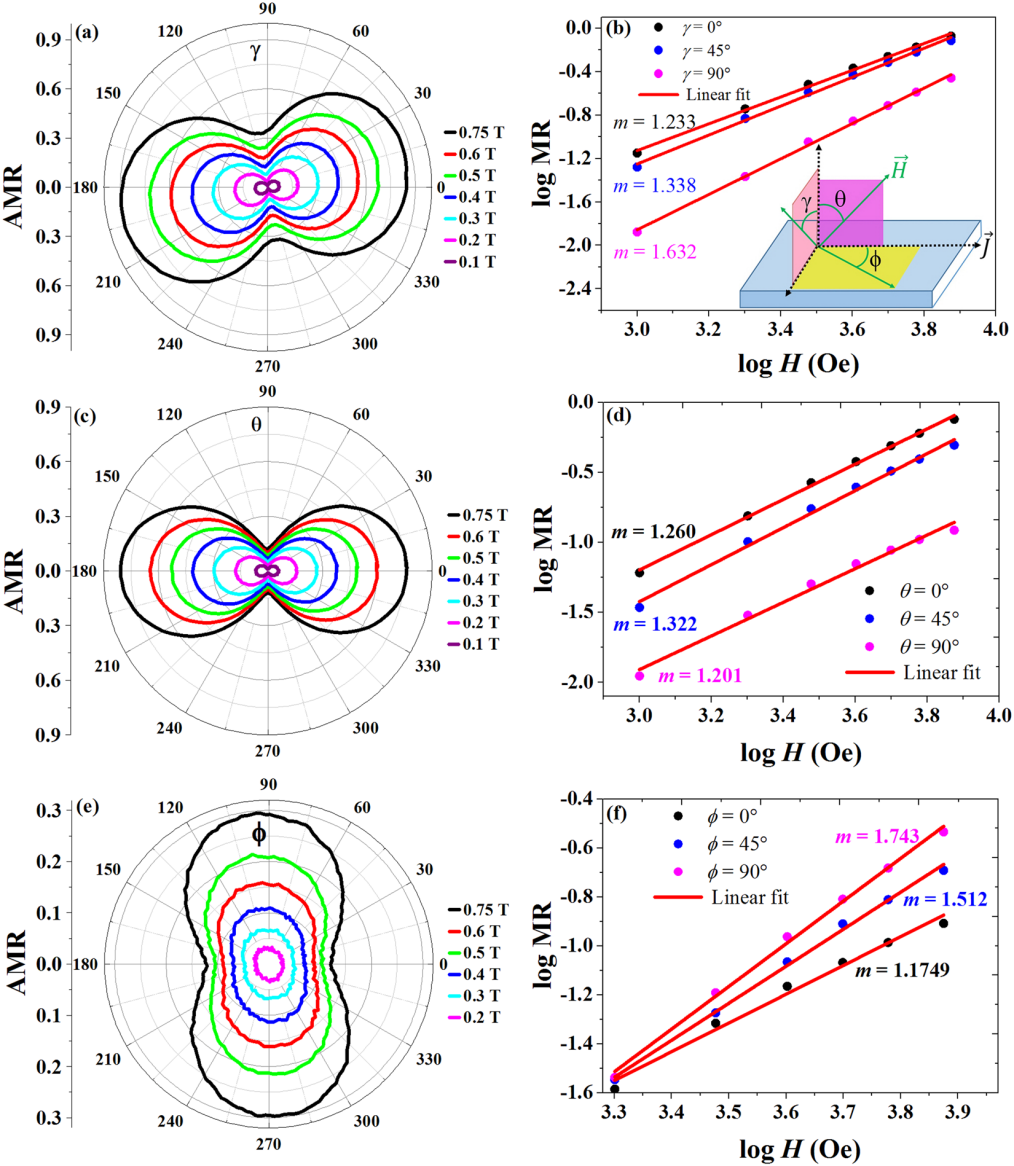
(**a**,**c**,**e**) show the polar plots of anisotropic magnetoresistance (AMR) for different magnetic field strengths from 0.1 T to 0.75 T at 6 K for *γ*, *θ* and *ϕ* orientations, respectively exhibiting the two-fold symmetry. (**b**,**d**,**f**) Plots of log (M.R.) vs log (H) shows the linear behavior. The solid lines are the linear fittings giving the slope (*m*) value, giving the order for field dependence. The insets show the definitions of *γ*, *θ* and *ϕ* within the measurement geometry.

**Figure 4:**
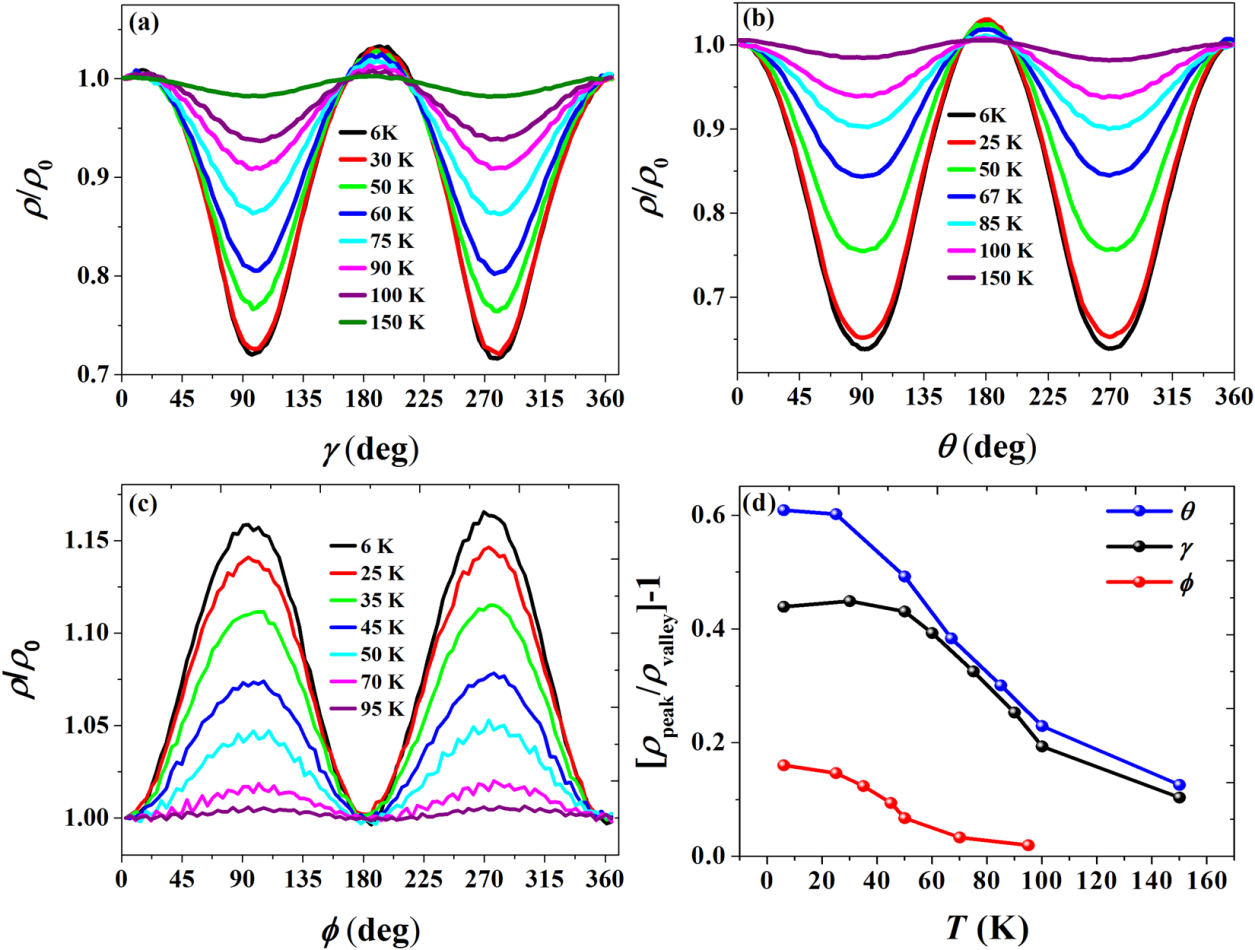
(**a**–**c**) show the normalized plots of temperature dependence of AMR measured at fixed magnetic field 0.75 T for *γ* (**a**), *θ* (**b**) and *ϕ* (**c**) orientations, respectively showing absence of phase transition up to the measured temperature range. (**d**) It shows the comparative plots of temperature dependence of magnetoresistance for three different orientations.

The anisotropic magnetoresistance (AMR) is measured along three different field orientations of *γ*, *θ* and *ϕ* at different field strengths from 0.1 T to 0.75 T as shown in Fig. 3(a,c,e). The inset of Fig. 3(b) shows the AMR measurement geometry for the *γ*, *θ* and *ϕ* orientations. The AMR effect for the *γ* orientation is presented in Fig. 3(a) as a polar plot, which illustrates the two-fold symmetry with a variation of period π. In this configuration, the magnitude of AMR reaches a maximum about 75% at γ = 10° and minium about 32% at γ = 100° for the field of 0.75 T and temperature of 6 K, and a similar trend continues in the opposite way up to 180°. In the *ϕ* orientation, the magnitude of AMR shows a minimum about 12% at *ϕ* = 0° when the current is parallel to *H*, and maximum about 29.5% at *ϕ* = 90*°* when the current is perpendicular to H. The magnitudes of AMR are observed to be 75% and 12% for *I*⊥*H* (*θ* = 0°) and *I*∥*H* (*θ* = 90°), respectively, in the *θ* orientation. From the field dependent AMR measurements in three different orientations, it is clear that the AMR is positive, and its maximum always appears when the current is perpendicular to *H*. In order to analyse the power law dependence of MR, a double-logarathmic value between *H* and MR was taken for *γ*, *θ* and *ϕ* orientations as shown in Fig. 3(b,d,f), and the linear fitting of these plots yield the different slopes (m values) at various angles. The slope (m) varies from 1.233 at 0° to 1.632 at 90° for *γ* orientation. In the *θ* orientation, m values are found to be 1.260 at *θ* = 0° (*I*⊥*H*) and 1.201 *θ* = 90° (*I*∥*H*). For in-plane orientation (*ϕ*), m varies from 1.175 at *ϕ* = 0° to m = 1.743 at *ϕ* = 90°. It is noteworthy that the power law dependence of MR is close to 1 particularly at *ϕ* = 0° and *θ* = 90° with *I*∥*H* orientation. We also remark that the behavior of AMR with two-fold symmetry for *γ* and *θ* orientations remains the same regardless of the magnetic field strengths up to 0.75 T, whereas, for *ϕ* orientation, the two-fold symmetry in AMR gradually faded away in low field regime. The response of the charge carriers to the rotating magnetic field of magnitude about 0.75 T for three different orientations is studied as a function of temperature as shown in Fig. 4(a–c). The variation of AMR, ${\rm{\Delta }}\rho 1=[{\rho }_{peak}-{\rho }_{valley}]/{\rho }_{valley}$, with respect to temperature is presented in Fig. 4(d) for three different orientations. From the temperature dependence of magnetoresistance, AMR increases with decreasing temperature, and it is almost saturated at low temperatures. The two-fold symmetry is well pronounced at low temperature (6 K), and it is sustained up to a measured temperature of 150 K.

**Figure 5:**
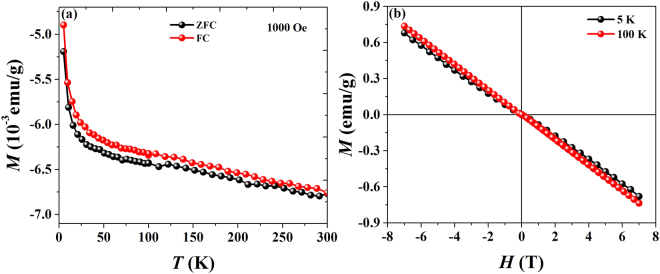
(**a**,**b**) show the *M-T* for 1000 Oe and *M-H* curves for 5 K and 100 K revealing the diamagnetic behavior in NbAs_2_.

Since the MR value of conventional metals is usually small in magnitude and saturated at high fields, and the consequences of unsaturated XMR and ultrahigh mobility in nonmagnetic topological semimetals such as Cd_3_As_2_^16^, TaAs, etc, is related to Dirac and Weyl fermions (topological surface states and linear band dispersion), the fact that NbAs_2_ exhibits unsaturated XMR is extremely important. In order to identify the intrinsic magnetic property of NbAs_2_, magnetization measurements as a function magnetic field and temperature were carried as shown in Fig. 5(a,b). The linear field dependence of magnetization in NbAs_2_ is similar to that observed for graphite^19,20^. Even though a sudden rise of magnetization below 25 K due to small amount of magnetic impurities, there is no significant effect in the AMR behavior of NbAs_2._

In ferromagnetic metals, AMR typically shows the maximum resistivity when the current is parallel to magnetic field due to spin-orbit scattering, and minimum resisitivity when the current is perpenducular to magnetic field^21,22^. Since the NbAs_2_ belongs to nonmagnetic material category, the physical origin of the AMR effect in the present system is thus different from that in magnetic materials.

According to the semiclassical two-band model^23,24^, the total conductivity tensor is expressed in the complex form of

2 $$\hat{\sigma }=e[\frac{n{\mu }_{n}}{(1+i{\mu }_{n}H)}+\frac{p{\mu }_{p}}{(1-i{\mu }_{p}H)}],$$

where the *n*(*p*) and *μ*_*n*_(*μ*_*p*_) are electron (hole) concentration and electron (hole) mobility, respectively; *e* is the electron charge and ‘*H*’ is the magnetic field. The total conductivity is then expressed as

3 $$\hat{\sigma }=e[\{\frac{n{\mu }_{n}}{(1+{\mu }_{n}^{2}{H}^{2})}+\frac{p{\mu }_{p}}{(1+{\mu }_{p}^{2}{H}^{2})}\}+i\{\frac{-n{\mu }_{n}^{2}H}{(1+{\mu }_{n}^{2}{H}^{2})}+\frac{p{\mu }_{p}^{2}H}{(1+{\mu }_{p}^{2}{H}^{2})}\}]$$

In equation (2), the Re and Im $\hat{\sigma }$ equal to ${\sigma }_{xx}$ and ${\sigma }_{xy}$, respectively where ${\sigma }_{xy}=\,\frac{{\rho }_{xy}}{{\rho }_{xy}^{2}+{\rho }_{xx}^{2}}\,$and ${\sigma }_{xx}=\,\frac{{\rho }_{xx}}{{\rho }_{xy}^{2}+{\rho }_{xx}^{2}}\,$and *ρ*_*xy*_ and *ρ*_*xx*_ are Hall and transverse resistivities, respectively. The magnetoresistance (MR) then follows

4 $$MR=\frac{\rho (H)-\rho (0)}{\rho (0)}=\frac{n{\mu }_{n}p{\mu }_{p}{({\mu }_{n}+{\mu }_{p})}^{2}{H}^{2}}{{(n{\mu }_{n}+p{\mu }_{p})}^{2}+{((n-p){\mu }_{n}{\mu }_{p}H)}^{2}}$$

**Figure 6:**
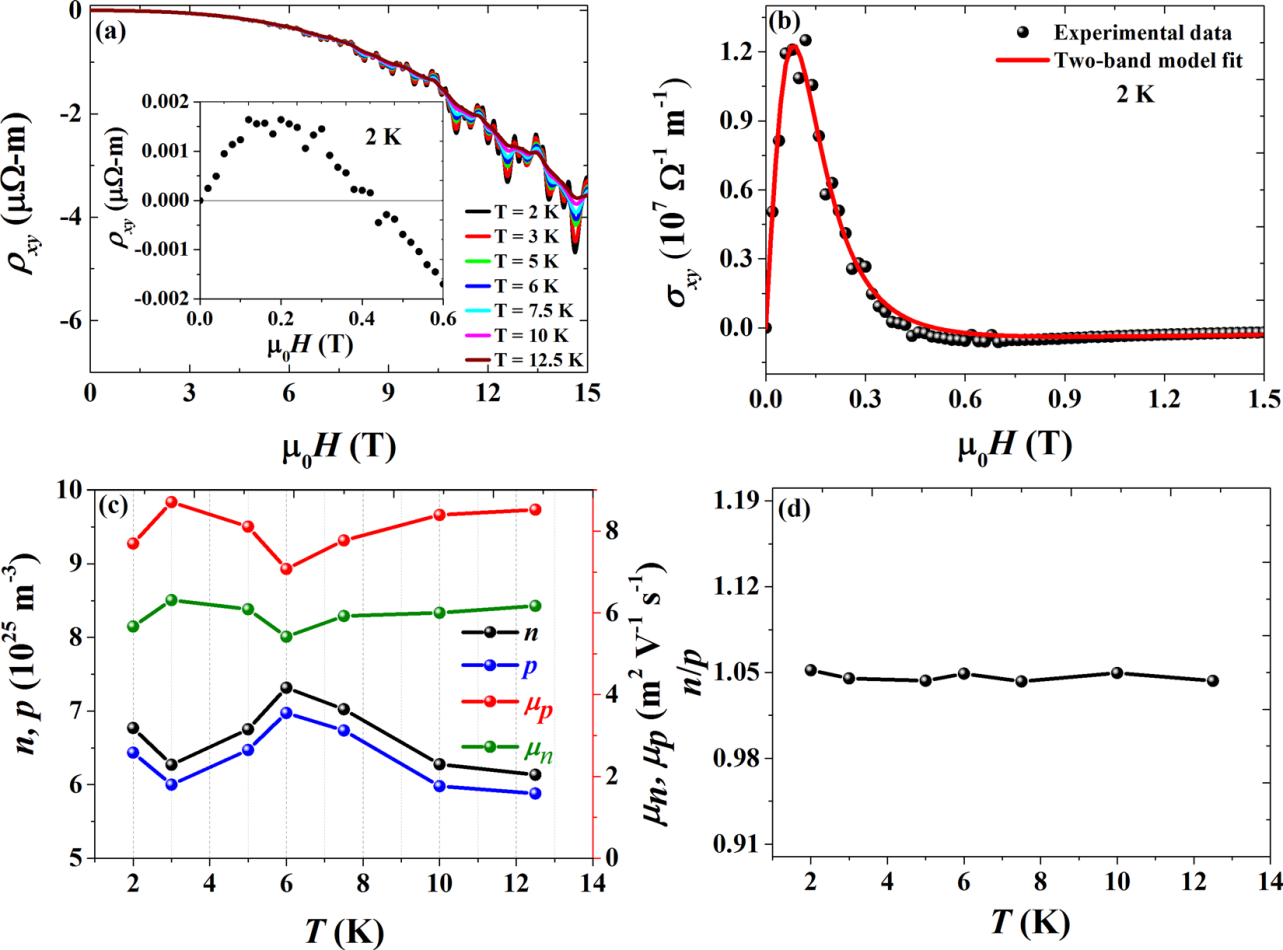
(**a**) Field dependence of the Hall resistivity (*ρ*_*xy*_) at various temperatures. Inset shows the expanded view of *ρ*_*xy*_ in the low field region of about 0.6 T at 2 K. (**b**) Field dependence of the Hall conductivity at 2 K. The solid line is the fit of the two-band model giving the carrier concentrations and mobilities of electrons and holes. (**c**) Plot of carrier concentrations and mobilities of electrons and holes as a function of temperature. (**d**) The ratio of n to p as a function of temperature demonstrates the electron-hole compensation.

From equation (2) *n*_,_ *p*, *μ*_*n*_ and *μ*_*p*_ can be obtained by fitting the *σ*_*xy*_(*H*) data. For a perfect compensated system (*n* = *p*), MR follows a quadratic field dependence which is shown in Fig. 2(b). Figure 6(a) shows the Hall resistivity as a function of magnetic field with nonlinear behavior at low temperatures. The expanded view of *ρ*_*xy*_ below 1 T of *H* at 2 K is shown in the inset of Fig. 6(a). It is clear that the sign of *ρ*_*xy*_ changes from positive (*ρ*_*xy*_ > 0 in H < 0.4 T) to negative at high fields with nonlinear band, suggesting the multiband effect in the NbAs_2_ system. At 200 K, *ρ*_*xy*_ shows positive value below 8 T of magnetic field as shown in Fig. S1(c) which suggests that holes dominate over electrons in the transport properties. By fitting the *σ*_*xy*_ data as shown in Fig. 6(b), carrier concentrations of *n* = 6.7691 × 10^25^ m^−3^ and *p* = 6.4352 × 10^25^ m^−3^, and mobilities of *μ*_*p*_ = 7.6947 m^2^ V^−1^ s^−1^ and *μ*_*n*_ = 5.6676 m^2^ V^−1^ s^−1^ at 2 K are extracted, as shown in Fig. 6(c). Figure 6(d) shows the ratio of *n* to *p* as the function of temperatures, suggesting that these two carriers are almost compensated in the NbAs_2_ system.

The magnetic field and temperature dependent transport measurements revealed the highly compensated electron and hole pockets, which may be responsible for the observed XMR. Recently, the AMR effect is observed in several nonmagnetic materials such as ZrSiS^25^, LaBi^26^, WTe_2_^27^, etc,. For example, the AMR effect with the combination of two and four-fold symmetry and unsaturated MR with electron-hole compensation as well as open orbital of Fermi surface had been reported in a Dirac semimetal ZrSiS^28^. The large AMR may be regarded as the most promiment signature in transport for the non-zero Berry curvatures in topological systems^29^. The transport features of NbAs_2_ we observed turn out to be similar to the Dirac semimetal ZrSiS^25^ and WTe_2_^27^. Further theoretical calculations and band structure characterizations are keenly required to reveal the possible nontrivial band topology in NbAs_2_.

In summary, the single crystals of NbAs_2_ were grown using the chemical vapour transport method. We observed extremely large, unsaturated and anisotropic MR in NbAs_2_. Transverse magnetoresistance of NbAs_2_ reaches a large value of about 303,200% at 2 K and 15 T, and MR follows a quadratic field dependence, which is in accord with the electron-hole compensation with the n/p ratio of about 1.05 determined from semiclassical two-band model fittings. From the SdH quantum oscillations, two distinct Fermi pockets were identified, and its effective electron mass and Dingle temperature were extracted from the L-K fitting. Interestingly, apparent two-fold symmetry and large magnitude in AMR are observed for three different field orientations, and power law dependence of MR is close to 1 for *I*∥*H* orientation. The origin of such large AMR effect in a non-magnetic semimetal NbAs_2_ may be related to the presence of non-trivial Berry curvature in NbAs_2_, where the magnetic contribution to the AMR effect has been excluded based on magnetization measurements.

## Experimental Section

### Sample preparation

Two step chemical vapor transport processes were used to synthesize and grow single crystals of NbAs_2_. A quartz ampoule with a length of 30–40 cm was used for the synthesis and growth. At first, stoichiometric amounts of 5 N purity precursors of Nb and As in a molar ratio of 1:2 were sealed in an evacuated quartz ampoule. The vacuum-sealed quartz ampoule containing the binary mixture was treated at 950 °C for two days and then cooled to room temperature, yielding polycrystalline NbAs_2_. Secondly, the polycrystalline powder of NbAs_2_ was mixed with I_2_ in a weight ratio of 100:1 and vacuum-sealed in a two-zone tube furnace having a thermal gradient of about 950-850 °C within ~40 cm. The resulting NbAs_2_ single crystals have shiny surfaces with well-defined crystal facets as shown in the inset of Fig. 1(b).

## Electronic supplementary material

Supplementary Information

## References

[1] Baibich MN (1988). Giant Magnetoresistance of (001)Fe/(001)Cr Magnetic Superlattices. Phys. Rev. Lett..

[2] Binasch G, Grünberg P, Saurenbach F, Zinn W (1989). Enhanced magnetoresistance in layered magnetic structures with antiferromagnetic interlayer exchange. Phys. Rev. B.

[3] Daughton JM (1999). GMR applications. J. Magn. Magn. Mater..

[4] Wang K, Graf D, Li L, Wang L, Petrovic C (2014). Anisotropic giant magnetoresistance in NbSb2. Sci. Rep..

[5] Zeng L-K (2016). Compensated Semimetal LaSb with Unsaturated Magnetoresistance. Phys. Rev. Lett..

[6] Lei SS, Q. W, P.-J. G, K. L, H. (2016). Large magnetoresistance in LaBi: origin of field-induced resistivity upturn and plateau in compensated semimetals. New J. Phys..

[7] Singha R, Pariari AK, Satpati B, Mandal P (2017). Large nonsaturating magnetoresistance and signature of nondegenerate Dirac nodes in ZrSiS. Proc. Natl. Acad. Sci..

[8] Zhang C-L (2017). Electron scattering in tantalum monoarsenide. Phys. Rev. B.

[9] Shekhar C (2015). Extremely large magnetoresistance and ultrahigh mobility in the topological Weyl semimetal candidate NbP. Nat Phys.

[10] Furuseth S, Kjekshus A (1965). The crystal structures of NbAs2 and NbSb2. Acta Crystallogr..

[11] Shen B, Deng X, Kotliar G, Ni N (2016). Fermi surface topology and negative longitudinal magnetoresistance observed in the semimetal NbAs2. Phys. Rev. B.

[12] Yuan Z, Lu H, Liu Y, Wang J, Jia S (2016). Large magnetoresistance in compensated semimetals TaAs2 and NbAs2. Phys. Rev. B.

[13] Wang Y-Y, Yu Q-H, Guo P-J, Liu K, Xia T-L (2016). Resistivity plateau and extremely large magnetoresistance in NbAs2 and TaAs2. Phys. Rev. B.

[14] Ronning NJG (2015). Magnetotransport of single crystalline NbAs. J. Phys. Condens. Matter.

[15] Ali MN (2014). Large, non-saturating magnetoresistance in WTe2. Nature.

[16] Liang T (2015). Ultrahigh mobility and giant magnetoresistance in the Dirac semimetal Cd3As2. Nat Mater.

[17] Tafti FF, Gibson QD, Kushwaha SK, Haldolaarachchige N, Cava RJ (2016). Resistivity plateau and extreme magnetoresistance in LaSb. Nat Phys.

[18] Shoenberg, D. *Magnetic Oscillations in Metals*. (Cambridge University Press, 1984).

[19] Ramos MA (2010). Magnetic properties of graphite irradiated with MeV ions. Phys. Rev. B.

[20] Li Z (2015). Field and temperature dependence of intrinsic diamagnetism in graphene: Theory and experiment. Phys. Rev. B.

[21] McGuire T, Potter R (1975). Anisotropic magnetoresistance in ferromagnetic 3d alloys. IEEE Transactions on Magnetics.

[22] Rushforth AW (2007). Anisotropic Magnetoresistance Components in (Ga,Mn)As. Phys. Rev. Lett..

[23] Ziman, J. M. *Electrons and Phonons: The Theory of Transport Phenomena in Solids*. (Oxford University Press, 2001).

[24] N W. Ashcroft, N. D. M. *Solid State Physics*. (Saunders College, 1976).

[25] Ali, M. N. *et al*. Butterfly magnetoresistance, quasi-2D Dirac Fermi surface and topological phase transition in ZrSiS. *Sci*. *Adv*. **2**, (2016). 10.1126/sciadv.1601742 PMC5161428 28028541

[26] Kumar N (2016). Observation of pseudo-two-dimensional electron transport in the rock salt-type topological semimetal LaBi. Phys. Rev. B.

[27] Zhao Y (2015). Anisotropic magnetotransport and exotic longitudinal linear magnetoresistance in WTe_2_ crystals. Phys. Rev. B.

[28] Lv Y-Y (2016). Extremely large and significantly anisotropic magnetoresistance in ZrSiS single crystals. Appl. Phys. Lett..

[29] Nandy S, Sharma G, Taraphder A, Tewari S (2017). Chiral Anomaly as the Origin of the Planar Hall Effect in Weyl Semimetals. Phys. Rev. Lett..

