# Supplementary material for: Anisotropic magnetotransport and extremely large magnetoresistance in NbAs2 single crystals
Source: Sci Rep. 2018 Apr 23;8:6414. doi: 10.1038/s41598-018-24823-z (PMC5913139; doi:10.1038/s41598-018-24823-z)
Supplement: Supplementary file 1 — Supplementary Information [file 41598_2018_24823_MOESM1_ESM.docx]

**Anisotropic magnetotransport and extremely large magnetoresistance in NbAs_2_ single crystals**

G. Peramaiyan ^1^, Raman Sankar^1,2*^, I. Panneer Muthuselvam ^1,2,3^, Wei-Li Lee^1^

^1^Institute of Physics, Academia Sinica, Taipei 10617, Taiwan

^2^Center for Condensed Matter Sciences, National Taiwan University, Taipei 10617, Taiwan

^3^ Department of Materials Science, Central University of Tamil Nadu, Neelakudi, Thiruvarur-610005, Tamil Nadu, India

Figure S1(a) presents the temperature dependence of resistivity for different static magnetic fields (*H*) (1T – 7 T) in the direction of *H* perpendicular to the electric current *I* for sample 2*.* At *H* = 0, NbAs_2_ exhibits a metallic profile with *ρ_xx_* (300 K) = 160 μΩ cm showing strong linear temperature dependence up to 85 K and weak temperature dependence down to 5 K. When the magnetic field is applied, a metal-insulator-like transition occurs in the temperature dependent resistivity particularly in the low temperature region. The resistivity decreases monotonically up to a minimum temperature *T*_m_ below which the *ρ_xx_*(*T*) starts to increase until an inflection at *T_i_* where it attains saturation followed by a plateau. The similar behavior of metal-insulator-like cross-over has been observed in the different classes of semimetallic systems with large magnetoresistance (MR). Fig.S1(b) shows the field dependent resistivity with parabolic behavior for different temperatures in the ***I*⊥*H*** configuration and the quadratic field dependence is clearly seen in Fig.S1(d). The hallmark of material with nonlinear behavior in field dependent resistivity is the multiband effect, which is clearly seen in Fig.S1(c). Fig.S1(e) shows the logarithmic plot between MR and *H* for different temperatures. The slope values (m) obtained from the linear fiiting reveal the quadratic field dependence, *m* = 1.86 at 2 K, and the same trend continues up to 200 K with *m* = 1.74.

**Figure S1 (a) Temperature dependence of resistivity at various magnetic fields for sample 2, showing metal-insulator-like transition. (b) Field dependence of resistivity *(ρ_xx_)* of sample-2 in the *I*⊥*H* configuration at various temperatures. (c) Field dependence of the Hall resistivity at various temperatures shows nonlinear trend, suggesting multiband effect in the NbAs_2_ system. (d) Quadratic field dependence of MR at various temperatures reveals electron-hole compensation. (e) Plot of log *H* vs log MR shows the slope (*m*) values for different temperatures, revealing quadratic field dependence. The red solid line is the linear fitting.**

**Figure S2 (a) shows the reconstructed oscillatory pattern for *I*⊥*H* direction. (b) Filtered resistivity oscillatory pattern (*δρ_xx_*) for the frequency 266 T.**

From the resistivity oscillatory pattern (*∆𝛒_xx_*), two distinct frequency peaks F_α_ = 266 T and F_β_ = 32 T were observed for *I*⊥*H* direction. Since the pure oscillatory pattern seems complex, we used band pass FFT filter to separate the oscillations for the each of the observed frequencies. Since the filtered oscillatory pattern for 32 T is not periodic, it is not possible to find its effective cyclotron mass. Using the separated oscillatory patterns (δρ_xx_), effective cyclotron mass was extracted.

**Figure S3 (a), (c)&(e) show the anisotropic magnetoresistance (AMR) for different magnetic field strengths from 0.1 T to 0.75 T at 6 K for γ, θ and ϕ orientations, respectively. The insets show the definitions of γ, θ and ϕ within the measurement geometry. (b), (d)&(f) show the anisotropic magnetoresistivity for different temperature with magnetic field fixed at 0.75 T.**

**Figure S4 (a), (b)&(c) show the magnetic field dependence of magnetoresistance (MR) at 6 K for *γ*, *θ* and *ϕ* orientations, respectively.**
